# No Correlation between TIMP2 -418 G>C Polymorphism and Increased Risk of Cancer: Evidence from a Meta-Analysis

**DOI:** 10.1371/journal.pone.0088184

**Published:** 2014-08-19

**Authors:** Raju K. Mandal, Naseem Akhter, Shafiul Haque, Aditya K. Panda, Rama D. Mittal, Mohammed A. A. Alqumber

**Affiliations:** 1 Department of Urology, Sanjay Gandhi Post Graduate Institute of Medical Sciences, Lucknow, Uttar Pradesh, India; 2 Department of Laboratory Medicine, Faculty of Applied Medical Sciences, Albaha University, Albaha, Saudi Arabia; 3 Department of Biosciences, Jamia Millia Islamia (A Central University), New Delhi, India; 4 Department of Infectious Disease Biology, Institute of Life Sciences, Bhubaneswar, Odisha, India; University of Colorado, Denver, United States of America

## Abstract

**Aim:**

Tissue inhibitor of metalloproteinase (TIMP2) is involved in the regulation of matrix metalloproteinase 2 (MMP2) and shown to implicate in cancer development and progression. The results from the published studies based on the association between TIMP2 -418 G>C polymorphism and cancer risk are inconsistent. In this meta-analysis, we aimed to evaluate the potential association between TIMP2 -418 G>C polymorphism and cancer risk.

**Methodology:**

We searched PubMed (Medline) and EMBASE web databases to cover all studies based on relationship of TIMP2 -418 G>C polymorphism and risk of cancer until October 2013. The meta-analysis was performed for selected case-control studies and pooled odds ratios (ORs) and 95% confidence intervals (95% CIs) were calculated for all genetic models.

**Results:**

A total of 2225 cancer cases and 2532 controls were included from ten eligible case-control studies. Results from overall pooled analysis suggested no evidence of significant risk between TIMP2 -418 G>C polymorphism and cancer risk in any of the genetic models, such as, allele (C vs. G: OR = 1.293, 95% CI = 0.882 to 1.894, p = 0.188), homozygous (CC vs. GG: OR = 0.940, 95% CI = 0.434 to 2.039, p = 0.876), heterozygous (GC vs. GG: OR = 1.397, 95% CI = 0.888 to 2.198, p = 0.148), dominant (CC+GC vs. GG: OR = 1.387, 95% CI = 0.880 to 2.187, p = 0.159) and recessive (CC vs. GG+GC: OR = 0.901, 95% CI = 0.442 to 1.838, p = 0.774) models. No evidence of publication bias was detected during the analysis.

**Conclusions:**

The present meta-analysis suggests that the TIMP2 -418 G>C polymorphism may not be involved in predisposing risk factor for cancer in overall population. However, future larger studies with group of populations are needed to analyze the possible correlation.

## Introduction

Cancer is a multifactorial disease which results from complex interactions between various genetic and environmental factors [1], it remains a major global health problem and lead to increased morbidity and mortality worldwide [2]. The precise etiology of this deadly disease is also unclear. The most common form of genetic variation, i.e., single nucleotide polymorphisms (SNPs) is known to contribute individual susceptibility to cancer through interaction with environmental factors [3]. Therefore, it is anticipated that the identification of host genetic factors for susceptibility to cancer would greatly assist the global control and therapeutic strategies of this lethal disease.

Tissue inhibitor of matrix metalloproteinase (TIMP2, located at 17q25) is a secretory protein, which inhibits the proteolytic activity of matrix metalloproteinase 2 (MMP2), a member of protease family principally involved in the degradation of the extracellular matrix (ECM) [4]. Additionally, TIMP2 also regulates cell growth and apoptosis [5]. The balance between TIMP2 and MMP2 plays a significant role in maintaining the integrity of healthy tissues. The sequence variants within TIMP2 genes presumably disrupt this balance and are seemingly associated with the susceptibility for the development of tumor growth and progression. Low and high amounts of TIMP2 expression have been found to be associated with different types and metastasis of cancer and in several cases it has been shown to be associated with a poor patient prognosis [6]–[8]. A single nucleotide G>C (rs8179090) polymorphism has been identified at position -418 in the promoter region of the TIMP2 gene [9] and it is postulated that this variant may affect gene expression, perhaps influencing the binding of the Sp1 transcription factor on a consensus sequence in the promoter region of the TIMP2 gene [10].

Considering the vital role of TIMP2 in carcinogenesis, several molecular epidemiological case-control studies have been performed to investigate the possible association between the TIMP2 -418 G>C polymorphism and cancer susceptibility in various neoplasm in different populations [11]–[20]. Though, the findings were inconsistent and contradictory. Inconsistency in results of these studies could possibly be attributed to the ethnicity of the population or sample size from individual studies that have low power to evaluate the overall effect. Thus, it is necessary to quantify and summarize the results from all eligible studies with rigorous methods. In the present study, we performed the meta-analysis to evaluate the overall association of -418 G>C polymorphism in risk/resistance to the development of cancer. A meta-analysis is a powerful tool to derive precise conclusion from pooled data and mostly utilized for the investigation of the risk factors associated with genetic diseases. It employs quantitative method to combine the data from individual studies where individual sample sizes are small and have low statistical power [21], [22].

## Materials and Methods

### Identification and eligibility of relevant studies

This meta-analysis was organized and reported according to the Preferred Reporting Items for Systematic Reviews and Meta-Analyses (PRISMA) statement (Checklist S1). We searched electronic research literature from PubMed (Medline) and EMBASE web databases with the combination of following keywords: ‘TIMP2, Tissue inhibitor of metalloproteinase 2 gene (polymorphism OR mutation OR variant) AND cancer susceptibility or risk (last updated on October 2013). The search was focused on studies that had been conducted in human subjects. All retrieved articles were evaluated by reading the titles and abstracts, and all published studies matching with the above said eligible criteria were included in this meta-analysis. We also did manual search of reference list from the retrieved articles for other eligible research articles.

### Inclusion and exclusion criteria

Articles included in the present meta-analysis had to meet all the following criteria: a) must evaluated the association between TIMP2 (-418 G>C) polymorphism and risk of cancer, b) used a case-control study design and were conducted in human beings, c) published in the English language, d) have available genotype frequency in cases and controls. Inclusion criteria for cases and controls were, a) cancer cases should confirmed histologically or pathologically, b) controls should be healthy and free of any type malignancy, c) both, the controls and the cases should have similar ethnicity. Additionally, when the same patient population was appeared in more than one publication, then only the most recent or complete study was included in this meta-analysis. The main reasons for study exclusion were, overlapping of the data, case-only studies, review articles, and genotype frequencies or numbers of the subjects were not reported. The supporting information related to the selection procedure (showing inclusion and exclusion criteria) of the studies has been appended as Figure S1 (PRISMA 2009 Flow Diagram).

### Data extraction and quality assessment

For each retrieved publication, the methodological quality assessment and data extraction were independently abstracted in duplicate by two independent investigators employing a standard protocol. The data-collection form was used to ensure the accuracy of the collected data by strictly adopting the inclusion criteria as mentioned above. The main characteristics abstracted from the retrieved studies included the name of the first author, year of research publication, the country of origin, the number of cases and controls, type of cancer, genotype frequencies for cases and controls and source of genotyping. Cases related with conflict or disagreement on any item of the data from the collected studies was fully debated with investigators to attain a final consensus.

### Statistical analysis

In order to evaluate the strength of association between TIMP2 -418 G>C polymorphism and cancer risk, pooled ORs and their corresponding 95% CIs were calculated [23]. Heterogeneity assumption between studies across the eligible comparison was done by the chi-square-based Q-test [24]. Heterogeneity was considered significant at p-value <0.05 to avoid underestimation of the presence of heterogeneity. A fixed effect model (if p-value >0.05) [25] or a random effect model (if p-value <0.05) [26] was used for pooling the results. Also, I^2^ statistics was employed to efficiently test the heterogeneity [27]. Hardy-Weinberg equilibrium (HWE) in the controls was measured via chi-square test. Funnel plot asymmetry was measured by Egger's linear regression test which is a type of linear regression approach to estimate the funnel plot asymmetry on the natural logarithm scale of the OR. The importance of the intercept was determined by the t-test (p-value <0.05 was considered as representation of statistically significant publication bias) [28]. In order to select the most suitable program to perform the current meta-analysis, an online comparison of ‘meta-analysis’ software programs was carried out using uniform resource locator (url) address http://www.meta-analysis.com/pages/comparisons.html. The Comprehensive Meta-Analysis (CMA) version 2 software program from Biostat Inc., NJ, USA was utilized for performing all statistical analysis involved in the present meta-analysis. All p-values were two sided and statistical significance level was considered as p-value less than 0.05 for this meta-analysis.

## Results

### Literature search and meta-analysis databases

According to the inclusion and exclusion criteria, a total ten research articles were finally included in this meta-analysis through literature search from the PubMed (Medline) and EMBASE web databases. We excluded one article during our study selection procedure, which was published in the Chinese language [29]. All retrieved articles were scrutinized carefully by reading the titles and abstracts, and the full texts for the potentially relevant research articles were further examined for their appropriateness for this meta-analysis (Figure S1: PRISMA 2009 Flow Diagram). Studies either showing TIMP2 polymorphism to predict survival in cancer patients or considering TIMP2 variants as an indicators for response to therapy were excluded straightaway from this meta-analysis. Similarly, studies investigating the levels of TIMP2 mRNA or protein expression or relevant review articles were also excluded from this study. We included only case-control or cohort design studies having frequency of all three genotype. Besides the database search, the references available in the retrieved articles were also appraised for other potential articles (Table 1). Distributions of genotypes and minor allele frequency (MAF) in the controls and cases have been given in Table 2.

**Table 1:** Main characteristics of TIMP2 -418 G>C polymorphism included in this meta-analysis.

First Authors	Year	Country of origin	Cancer	Genotyping method	Cases	Controls	Source of genotyping
Zhou et al. [11]	2004	China	Breast	PCR-RFLP	462	509	Blood
Ocharoenrat et al. [12]	2006	Thailand	HNSCC	PCR-RFLP	239	250	Blood
Kuben et al. [13]	2006	Netherland	Gastric	PCR-RFLP	79	169	Tissue, Blood
Yang et al. [14]	2007	China	Gastric	PCR-RFLP	206	206	Blood
Vairaktaris et al. [15]	2007	Germany	OSCC	PCR-RFLP	158	168	Blood
Wu et al. [16]	2007	Taiwan	Gastric	PCR-RFLP	240	283	Blood
Yi et al. [17]	2009	Taiwan	Endometrial	PCR-RFLP	118	229	Blood
Park et al. [18]	2011	Korea	Colorectal	PCR-RFLP	333	318	Blood
Srivastava et al. [19]	2012	India	Prostate	PCR-RFLP	190	200	Blood
Srivastava et al. [20]	2013	India	Cervical	PCR-RFLP	200	200	Blood

Note: OSCC- Oral squamous cell carcinoma; HNCC- Head and neck squamous cell carcinoma; PCR - Polymerase chain reaction; RFLP- Restriction fragment length polymorphism.

**Table 2:** Genotypic distribution of TIMP2 -418 G>C gene polymorphism.

Authors and year	Control				Case				HWE
	Genotype			Minor allele	Genotype			Minor allele	
	GG	GC	CC	MAF	GG	GC	CC	MAF	p-value
Zhou et al. 2004	322	173	14	0.19	321	130	11	0.16	0.11
Ocharoenrat et al. 2006	174	66	10	0.17	147	90	2	0.19	0.24
Kuben et al. 2006	168	1	0	0.002	78	1	0	0.006	0.96
Yang et al. 2007	141	61	4	0.16	121	68	17	0.24	0.37
Vairaktaris et al. 2007	159	9	0	0.02	66	86	6	0.31	0.72
Wu et al. 2007	223	47	13	0.12	196	38	6	0.10	<0.0001
Yi et al. 2009	151	74	4	0.17	76	41	1	0.18	0.13
Park et al. 2011	185	115	18	0.23	223	97	13	0.18	0.98
Srivastava et al. 2012	144	54	2	0.14	147	43	0	0.11	0.21
Srivastava et al. 2013	165	35	0	0.08	150	49	1	0.12	0.17

### Publication bias

Begg's funnel plot and Egger's test were carried out to review the publication bias among the selected publications for the meta-analysis. The appearance of the shape of Begg's funnel plots was seemed symmetrical in all the genetic models of this study (Figure S2). The Egger's test was performed to suffice the statistical evidence of funnel plot. This test assesses the bias by using precision to predict the standardized effect. The Egger's test did not detect publication bias, but with 10 studies, there is little chance it could, and thus a non-significant test does not mean that there was no publication bias (Table 3).

**Table 3:** Statistics to test publication bias and heterogeneity in the meta-analysis.

Comparisons	Egger's regression analysis			Heterogeneity analysis			Model used for the meta-analysis
	Intercept	95% Confidence Interval	p-value	Q-value	P_heterogeneity_	I^2^ (%)	
C vs. G	4.13	−1.08 to 9.34	0.10	85.09	<0.0001	89.42	Random
CC vs. GG	0.73	−2.19 to 3.66	0.57	21.28	0.005	63.34	Random
GC vs. GG	4.47	−1.16 to 10.10	0.10	87.35	<0.0001	89.70	Random
CC+GC vs. GG	4.59	−1.16 to 10.35	0.10	93.09	<0.0001	90.41	Random
CC vs. GG+GC	0.39	−2.35 to 3.14	0.74	18.79	0.01	57.42	Random

### Evaluation of heterogeneity

In order to test heterogeneity among the selected publications, Q-test and I^2^ statistics were employed. Heterogeneity was observed in all the genetic models, i.e., allele (C vs. G), homozygous (CC vs. GG), heterozygous (GC vs. GG), dominant (CC+GC vs. GG) and recessive (CC vs. GG+GC). Thus, random effects model was applied to synthesize the data for this analysis (Table 3).

### Association of TIMP2 -418 G>C polymorphism and overall cancer susceptibility in all the population

**Figure 1:**
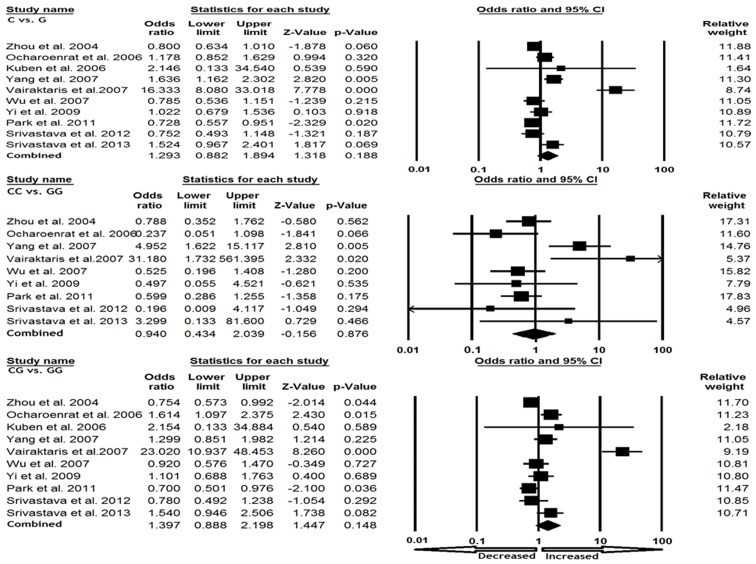
Forest plot with ORs on overall cancer risk associated with TIMP2 -418 G>C gene polymorphism. Note: The squares and horizontal lines correspond to the study-specific OR and 95% CI.

**Figure 2:**
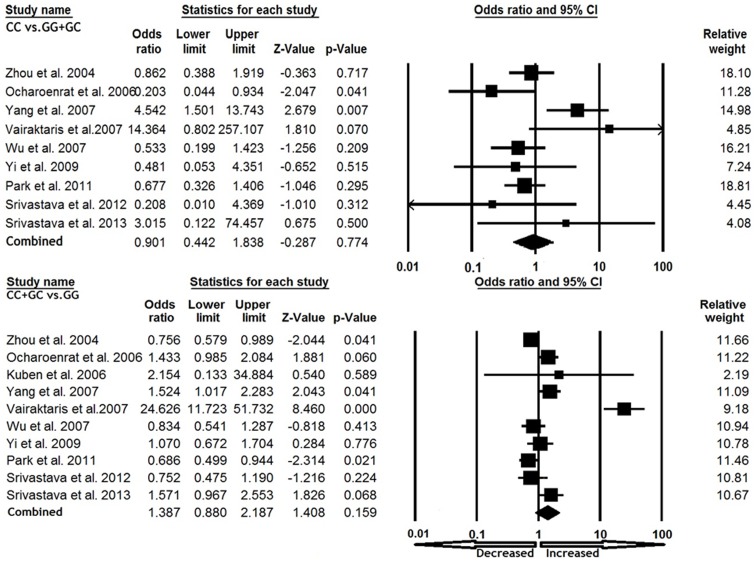
Forest plot with ORs on overall cancer risk associated with TIMP2 -418 G>C gene polymorphism. Note: The squares and horizontal lines correspond to the study-specific OR and 95% CI.

We pooled the data from all ten studies together and it yielded into 2225 cancer cases and 2532 control subjects, for appraisal of overall association between TIMP2 -418 G>C polymorphism and cancer risk. The pooled OR from overall studies indicated no significant increased or decreased risk between -418 G>C polymorphism and cancer susceptibility in allelic (C vs. G: OR = 1.293, 95% CI = 0.882 to 1.894, p = 0.188), homozygous (CC vs. GG: OR = 0.940, 95% CI = 0.434 to 2.039, p = 0.876), heterozygous (GC vs. GG: OR = 1.397, 95% CI = 0.888 to 2.198, p = 0.148), dominant (CC+GC vs. GG: OR = 1.387, 95% CI = 0.880 to 2.187, p = 0.159) and recessive (CC vs. GG+GC: OR = 0.901, 95% CI = 0.442 to 1.838, p = 0.774) comparison models (Figure 1 and 2).

### Sensitivity analysis

In order to evaluate the robustness of our meta-analysis results, we performed sensitivity analysis to determine whether the inclusion criteria of this meta-analysis affected the results or not [30]. Sequentially, single study was deleted each time to reflect the influence of individual data. The result of sensitivity analysis revealed that pooled ORs before and after the exclusion of the study were generally similar in all the five genetic models (Figure S3). Hence, results of the meta-analysis were relatively stable and credible.

## Discussion

The treatment and assessment of the risk and progression of cancer remains a grave problem, as it recurs despite of its removal via surgery, and chemotherapy does not decrease cancer incidence. It has been already established that genetic factors play an important role in the etiology of cancer and several low penetrance genes are involved in the progression of cancer. Therefore, genetic marker based risk assessment might offer some benefit to better predict the risk of cancer and early detection of the disease. TIMP2 hampers the growth of endothelial cells induced by basic fibroblast growth factor, thus suppressing angiogenesis and regulating apoptosis, indicates a negative role in cancer [31], [32]. TIMP2 possesses a complex role in cancer through its ability to regulate MMP activity and to inhibit especially MMP2. Angiogenesis is significant in carcinogenesis and proangiogenic factors play a key role in angiogenesis [33]. Given the important roles of TIMP2 in tumor growth, angiogenesis, invasion and metastasis, it is rational to speculate that host genomic polymorphism of TIMP2 may influence the tumor occurrence. In recent years, genetic variants of the TIMP2 gene and its role in the etiology of several types of cancer have been studied exhaustively, but the outcomes are inconclusive and contradictory. As it is known that, individual studies with a small sample size may have not sufficient statistical power to detect a small risk factor. In order to derive more precise results, we performed the current meta-analysis to assess whether an association exists between the TIMP2 gene polymorphism and risk of developing cancer. It has been established that pooled ORs generated from large sample size and population can increase the statistical power of the results and combining data from various studies has the advantage of reduced random errors [34].

To the best of our knowledge, this is the first meta-analysis study investigating the association between TIMP2 -418 G>C gene polymorphism and overall cancer risk. The associations for the allele contrast, dominant and recessive model were examined in the meta-analysis. The results indicated that -418 G>C polymorphism is not significantly associated with the susceptibility of cancer compared with GG genotype in overall populations. One of the possible explanations is that TIMP2 gene has several other SNPs and each polymorphism to cancer risk might be due to linkage disequilibrium (LD). It is well established that multiple SNPs perhaps act independently, collectively or interact with each other to influence the occurrence of cancer. Therefore, it is possible that multiple alleles or genes contribute the susceptibility to cancer risk and TIMP2 -418 G>C analyzed variant do not influence individually. Nevertheless, genetically complex diseases differ from simple Mendelian diseases and cancer etiology is polygenic, a single genetic variant is usually insufficient to predict the risk of this deadly disease that has a complex disease phenotype.

Heterogeneity is a crucial issue while interpreting the results of any meta-analysis study. Although, heterogeneity can be minimized by performing random-effects model [35]. In the present study we detected inter-study heterogeneity. There are several factors which contribute to such heterogeneity, for e.g., the genetic backgrounds for cases and controls, clinical characteristic of different tumors, diverse genotype distribution of TIMP2 in different ethnic groups and suggest that they are almost/always subject to natural selection [36]. In some of the selected studies, the controls were not uniformly defined and some studies included patient with benign disease which may contribute to the TIMP2 gene mutation and development of various cancers.

Despite the significant findings from our current analysis, we still have to acknowledge some of the limitations of this analysis. First, we only included studies published in English language, abstracted and indexed by the selected electronic databases were included for data analysis; it is possible that some pertinent articles published in other languages and/or indexed in some other electronic databases may have missed. Second, the result of this meta-analysis was based on unadjusted ORs because not all selected eligible studies stated adjusted ORs. Third, the role of gene-environment interactions and subgroup analysis were not considered in this study because of limited publications and insufficient data. In spite of this, our current meta-analysis has some advantage. First, there was no clear evidence of publication bias through qualitative funnel plot and quantitative Egger linear regression, which indicated that our results are statistically robust. Second, we used explicit criteria for study inclusion and performed strict data extraction and analysis to make satisfactory and reliable conclusion.

## Conclusion

In conclusion, a meta-analysis is an extremely valuable and powerful approach of data-analysis which pools both statistically significant and non-significant data from individual studies and generates a precise and absolute conclusion. The present meta-analysis evaluated the relationship of TIMP2 -418 G>C polymorphism with the risk of cancer and suggested that the TIMP2 -418 G>C polymorphism did not contribute increased or decreased risk of cancer. Further well designed large-scale studies with the consideration of gene-gene and gene-environment interactions should be encouraged to investigate the possible association. Here, we only analyzed the -418 G>C variant for cancer risk without considering the interaction between several other SNPs. In future, we will further explore the other pertinent interactions to facilitate the discovery of the pathogenesis of cancer.

## Supporting Information

Figure S1 **(PRISMA 2009 Flow Diagram): Flow chart displaying the identification and selection of the studies for the present meta-analysis.**
(TIF)

Figure S2 **Funnel plots of the Egger's test to detect publication bias in five different genetic models.** Each point represents a separate study. The OR was plotted on a logarithmic scale against the precision of the each study.
(TIF)

Figure S3 **Sensitivity analysis of TIMP2 -418 G>C polymorphism.**
(TIF)

Checklist S1 **(PRISMA 2009 Checklist).**
(DOC)
