# Supplementary figures and images for: No Correlation between TIMP2 -418 G>C Polymorphism and Increased Risk of Cancer: Evidence from a Meta-Analysis
Source: PLoS One. 2014 Aug 19;9(8):e88184. doi: 10.1371/journal.pone.0088184 (PMC4138026; doi:10.1371/journal.pone.0088184)

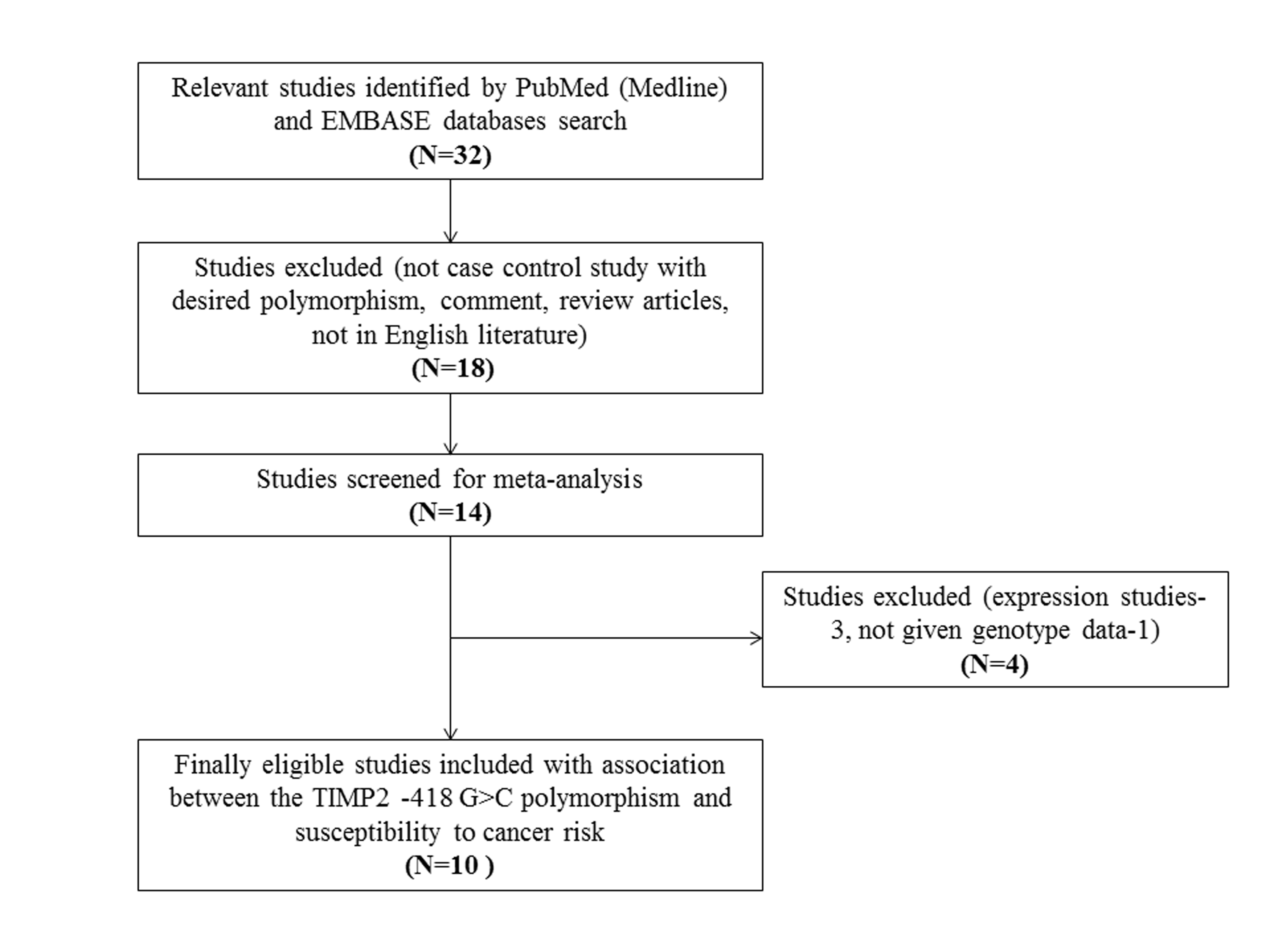

Supplement: Figure S1 — (PRISMA 2009 Flow Diagram): Flow chart displaying the identification and selection of the studies for the present meta-analysis. (TIF) [file pone.0088184.s001.tif]

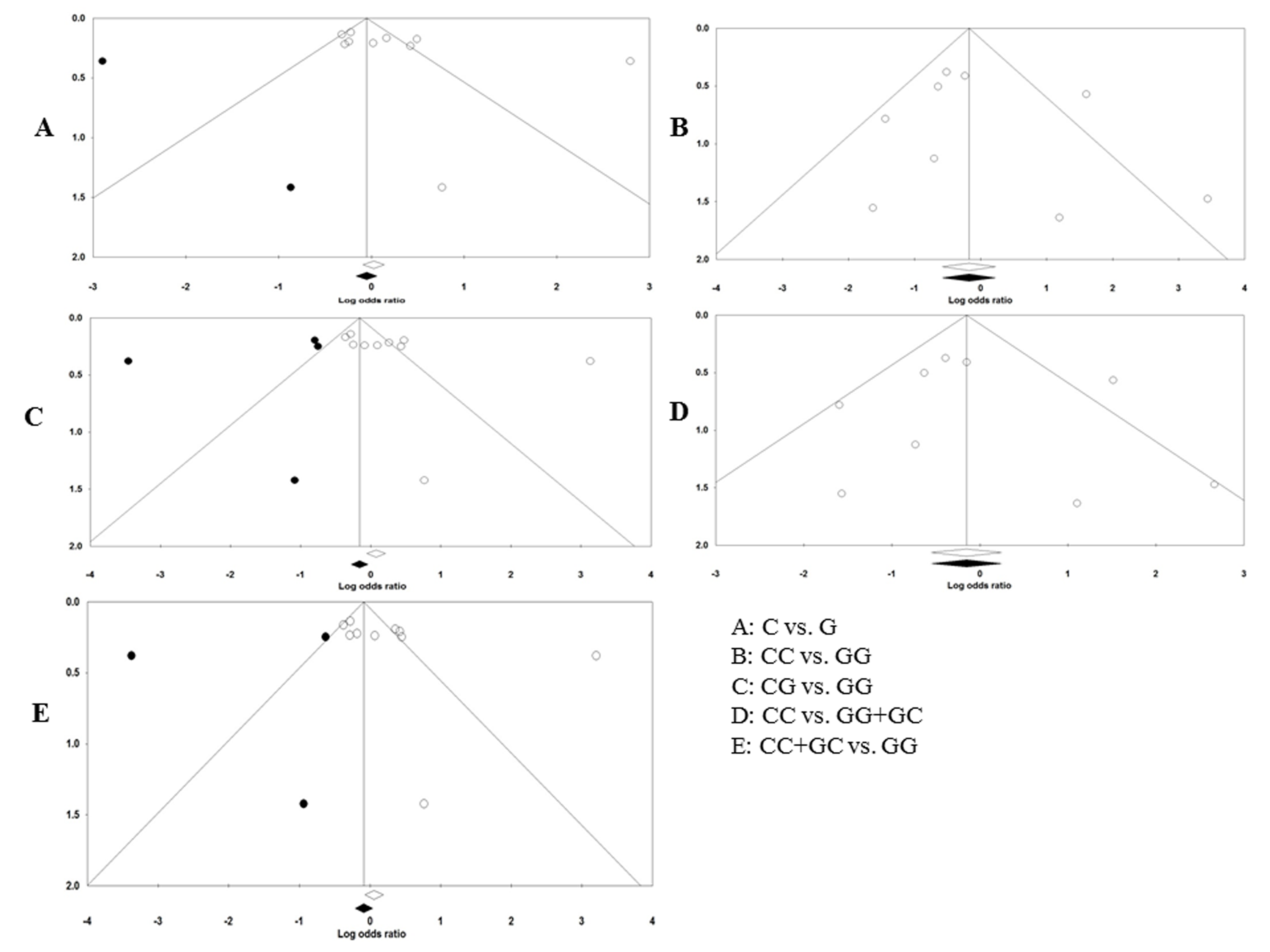

Supplement: Figure S2 — Funnel plots of the Egger's test to detect publication bias in five different genetic models. Each point represents a separate study. The OR was plotted on a logarithmic scale against the precision of the each study. (TIF) [file pone.0088184.s002.tif]

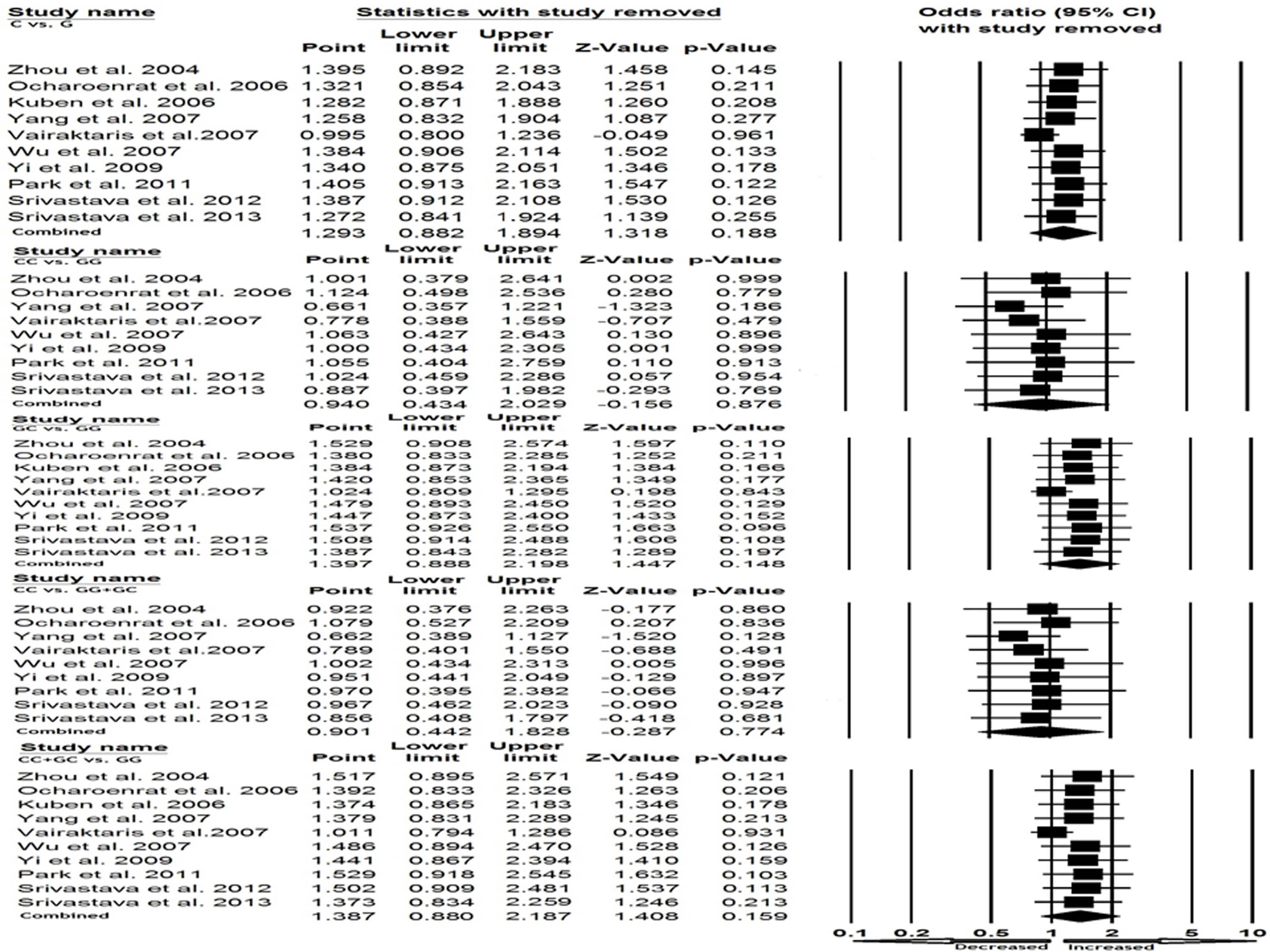

Supplement: Figure S3 — Sensitivity analysis of TIMP2 -418 G>C polymorphism. (TIF) [file pone.0088184.s003.tif]
